# Lacosamide Versus Propranolol in Episodic Migraine, a Randomized Controlled Double-blinded Trial

**DOI:** 10.1007/s12035-026-05756-5

**Published:** 2026-03-21

**Authors:** Mohamed G. Zeinhom, Sheren G. Rezk, Ahmed Mahmoud Mohamed, Sherihan Rezk ahmed

**Affiliations:** 1 Neurology Dep.,, Faculty of Medicine , Kafrelsheikh University, Kafrelsheikh, Egypt; 2 Pediatric Neurology Dep., , El-Eman Hospital, Assuit, Egypt; 3 Neurology Department, Damietta Hospital, Damietta, Egypt

**Keywords:** Episodic migraine, Egypt, Lacosamide, Propranolol

## Abstract

Although migraine is the second most prevalent form of headache, its preventive treatment has some contraindications and complications. It has been postulated that lacosamide might inhibit CGRP release in the trigeminal system, which could contribute to migraine management. We aimed to evaluate the efficacy and safety of lacosamide as an alternative medication to propranolol for preventing episodic migraine, especially in patients who cannot tolerate propranolol and approved antiseizure medicines for migraine prevention. We recruited patients with episodic migraines; we had two groups: lacosamide group who received lacosamide 50 mg once daily for 1 week, then twice daily from the 8th day till the 90th day; propranolol group who received propranolol 40 mg twice daily for 1 week, then 80 mg twice daily from the 8th till the 90th day. 574 completed the study. In episodic migraine patients, there was no significant difference in the MMD in the last 4 weeks of treatment compared to baseline, the percentage of patients who achieved > 50% reduction in migraine days, reduction in MMD that required acute medication in the last 4 weeks, reduction in HIT score compared to baseline in both groups with *P*-values 0.13, 0.22, 0.57, and 0.61 respectively. In episodic migraine patients, the regular use of lacosamide 50 mg Bid for 3 months yielded reductions in the monthly migraine days, migraine days that required acute medications, and HIT6 score comparable to those achieved using propranolol 80mg Bid. Lacosamide was well-tolerated by migraine patients. Trial registration: registered on clinicaltrial.gov, NCT05851781—30/04/2023.

**Supplementary Information:**

The online version contains supplementary material available at 10.1007/s12035-026-05756-5.

## Introduction

Migraine emerges as the second most prevalent form of headache after episodic tension-type headaches, carrying notable socioeconomic implications [1].

The global incidence of migraine is estimated to be as high as 14%, with a gender distribution ranging from 1:2 to 1:3, favouring females. The onset of migraine often occurs between the second and third decades of life, with prevalence increasing until the fourth decade, after which it declines [2].

The primary objectives of migraine therapy are to alleviate the overall impact and enhance quality of life by diminishing the frequency, intensity, and disability during and between migraine episodes, enabling patients to engage in their daily activities with standard functionality [3].

Anti-CGRP medications were first introduced in 2018. They were recommended in the case of failure of class I migraine preventive treatment and in chronic migraine patients. The recent guidelines for combining migraine preventive therapy recommend using oral migraine preventive treatment and anti-CGRP medications if there is an insufficient response to a single line of medicines, as they have different mechanisms of action [4].

Due to economic and logistical difficulties, anti-CGRP medications are still not widely available in many countries [5, 6], underscoring the need to evaluate other oral medications for migraine preventive therapy.

Although propranolol is a class I migraine preventive beta-blocker, it has some absolute contraindications in patients with atrioventricular block, heart failure, and bronchial asthma. It is relatively contraindicated in diabetes mellitus and autonomic dysregulation [7].

Lacosamide is a third-generation antiseizure amino acid molecule with the chemical formula R-2-acetamido-N-benzyl-3-methoxypropionamide, exhibiting two separate action modes. The first mechanism involves the delay of blockage of voltage-gated sodium channels (VGSCs), while the second mechanism involves their interaction with CRMP2 [8].

The neurovascular theory of migraine posited that alterations in nociceptive inputs originating from the raphe and locus coeruleus nuclei in the brainstem, or the occurrence of cortical spreading depression, can activate the trigeminal-vascular system in genetically susceptible individuals. This activation subsequently leads to migraine pain and its associated symptoms [9].

Calcitonin gene-related peptide (CGRP) is a highly consequential peptide released during migraine attacks, triggered by trigeminal-vascular system activation. Its release leads to vasodilation and inflammation in leptomeningeal and extracranial vessels, resulting in the pulsatile pain associated with migraines [9].

In the rat model, lacosamide has been shown to suppress CGRP release from brainstem explants under baseline conditions and following pharmacological stimulation and to reduce CGRP levels in the brainstem and trigeminal ganglia following nitroglycerin administration [10]. Another study showed that daily lacosamide 50 mg Bid for 3 months in patients with episodic migraine was associated with a significant reduction in serum CGRP-LI [11].

Our study aimed to evaluate the safety and efficacy of lacosamide as an antiseizure medication, potentially serving as a new alternative to propranolol for episodic migraine prevention.

## Methods

### Study Design

After receiving approval from the ethics council of the faculty of medicine at Kafr el-Sheikh University, we executed our double-blinded randomized controlled study and screened all episodic migraine patients diagnosed following ICHD-3 [12] and sought medical advice in Kafr-Elsheikh Hospital in the time from the 1 st of May 2022 to the 1 st of August 2025, the last patient was enrolled in our study on the 24th of April 2025, and in our study, we followed up with patients for 3 months.

In a one-to-one ratio, 600 migraineurs were randomly assigned to receive lacosamide or propranolol for 3 months.

## Participants

Our trial consisted of two parallel groups: the (A) group, which included 300 patients who received lacosamide, and the (B) group, which included 300 patients who received propranolol.

## Eligibility Criteria

The study recruited participants between the ages of 18 and 65 who were diagnosed with episodic migraines following ICHD-3 [13].

The detailed inclusion and exclusion criteria are available in the supplemental material.

## Interventions

We randomly assigned 600 patients to receive lacosamide or propranolol. All of our patients underwent routine laboratory tests and an MRI of the brain.

The study had two parallel groups: the (A) group, which consisted of 300 patients who received (lacosamide 50 mg once daily for 1 week, then twice daily from the 8th day till the 90th day) [11]; the (B) group, which consisted of 300 patients who received (propranolol 40 mg twice daily for 1 week, then 80 mg twice daily from the 8th day till the 90th day) [14].

The detailed study interventions are available in the supplemental material.

## Outcome Assessment

### Primary Outcome

The absolute change in migraine days in each group’s last 4 weeks of the treatment period (migraine day was defined as a calendar day when the patient reported four continuous hours of headache meeting ICHD-3 criteria for migraine).

### Secondary Outcomes

The percentage of patients who achieved ≥ 50% reduction in the baseline headache days frequency in the last 4 weeks of the treatment period [15], the absolute change in the number of migraine days in the last 4 weeks of the treatment period that required receiving acute headache medications compared to baseline, and the absolute change in the HIT-6 score in the last 4 weeks of the treatment period compared to baseline.

The secondary safety outcome was evaluated by monitoring and documenting treatment-emergent adverse events (TEAE) in patients through regular follow-up procedures for 3 months, including open-ended telephone conversations twice a week and face-to-face interviews in the outpatient clinic once a month.

### Sample Size

After using Power Analysis and Sample Size System (PASS, V12), NCSS), we determined that a total of 560 episodic migraine patients would provide 80% power to detect a mean difference of 1.5 migraine days per 4 weeks (primary outcome) in the lacosamide group as compared with the propranolol group, with a final two-sided significance level of 95%, alpha error of 5%, assuming the reduction in migraine days per 4 weeks is 4.6 ± 0.78 migraine days in the propranolol group [14] and an overall dropout rate of 5%. The final size of our trial was 600 patients, 300 patients in each group.

### Randomization and Blinding

Our study was a double-blinded trial.

The detailed study randomization and blinding are available in the supplemental material.

## Statistical analysis of the data

We used the IBM SPSS software package, version 29.0 (Armonk, NY: IBM Corp.) URL: https://www.ibm.com/support/pages/downloading-ibm-spss-statistics-29020, to analyze our data and base all efficacy and safety analyses on the intention-to-treat principle. Both the primary and secondary outcomes underwent separate statistical analyses. Depending on their distribution, as determined by the Shapiro–Wilk test, we reported numerical data as means ± S.D., medians with interquartile ranges (IQRs), or both. We also reported categorical data using numbers and percentages. The Mann–Whitney *U* test was used to compare irregularly distributed numerical data, while Pearson’s chi-square test was used to analyze categorical data. All statistical analyses were two-sided, and differences with a *P*-value of less than 0.05 were considered statistically significant. To avoid type 1 statistical error in the analysis of secondary efficacy outcomes, we used the Bonferroni correction for multiple comparisons, and differences with an adjusted *P*-value of less than 0.017 were considered statistically significant.

## Results

**Fig. 1:**
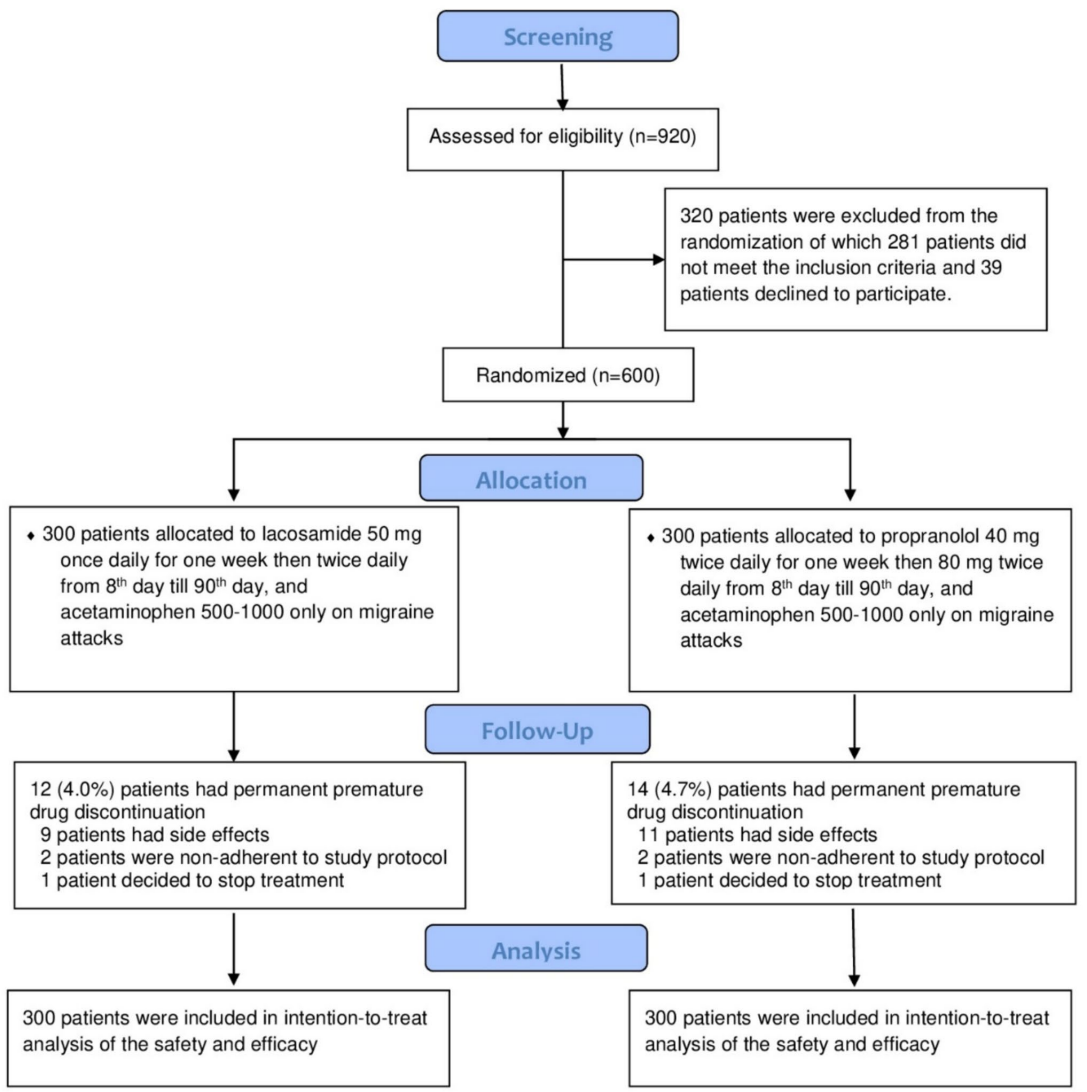
Study flow diagram

Nine hundred twenty patients were screened for eligibility; 320 patients were excluded from the study, of which 26 patients were known to be epileptics, 55 patients were lactating, 24 patients were pregnant, 18 patients had intracranial hemorrhage, 22 patients had malignancy, 32 had renal failure, 38 had liver cell failure, 40 patients had a contraindication to propranolol or lacosamide, 26 patients had primary headache other than migraine, and 39 patients declined to participate. Six hundred (131 males and 469 females) patients were enrolled in our study, of which 574 patients (120 males and 454 females) completed the 3-month follow-up period, as shown in Fig. 1.

As shown in Table 1, we did not find any statistically significant differences in baseline characteristics between the two arms of our trial.

**Table 1:** Baseline criteria of participants

Demographic data	Lacosamide arm (*n* = 300)	Propranolol arm (*n* = 300)
Age in years, Median (IQR)†	26.8 (14.7–40.5)	28.3 (16.4–40.0)
Sex*		
Female, no. (percentage)	239 (79.7%)	230 (76.7%)
Migraine characters†		
Baseline MMD, median (IQR)†	7.2 (7.0–10.0)	6.9 (7.0–10.0)
Disease duration in years, median (IQR)†	3.1 (2.0–5.0)	3.5 (2.0–5.5)
Attack duration in hours, median (IQR)†	7.5 (6.2–8.5)	7.0 (6.5–8.5)
HIT-6 score at baseline (median, IQR)†	47.3 (46.0–56.8)	46.8 (46.0–57.0)
Migraine days that required acute medications per 4 weeks before treatment, (median, IQR)†	4.5 (4.0–7.0)	4.6 (4.0–7*.0*)
Migraine site of maximal intensity†		
Frontal	140 (46.7%)	134 (44.7%)
Temporal	39 (13.0%)	51 (17.0%)
Parietal	26 (8.7%)	30 (10.0%)
Occipital	94 (31.3)	85 (28.3)
**Migraine pain location†**		
Holocranial	154 (51.3%)	175 (58.3%)
Hemicranial	49 (16.3%)	42 (14.0%)
Side locked	12 (4.0%)	9 (3.0%)
Side predominant	85 (28.3%)	74 (24.7%)
Migraine Subtypes*		
Migraine with aura, no. (percentage)	92 (30.7%)	82 (27.3%)
Migraine without aura, no. (percentage)	208 (69.3%)	218 (72.7%)
Migraine associated symptoms*		
Photophobia, no. (percentage)	249 (83.0%)	241 (80.3%)
Phonophobia, no. (percentage)	239 (79.7%)	231 (77.0%)
Nausea, no. (percentage)	231 (77.0%)	228 (76%)
Dizziness, no. (percentage)	204 (68.0%)	207 (69.0%)
Vomiting, no. (percentage)	120 (40.0%)	105 (35.0%)

†: Median (IQR, interquartile range);, *: Percentage, MMD,: monthly migraine days, HIT,: Headache Impact Test-6

Our trial showed that at the 90-day mark, the lacosamide group had a 2.58 ± 1.61-day reduction in monthly migraine days compared with baseline (*P*-value = 0.002). At the same time, the propranolol arm achieved a 2.67 ± 1.72-day reduction in monthly migraine days compared with baseline (*P*-value = 0.001).

**Fig. 2:**
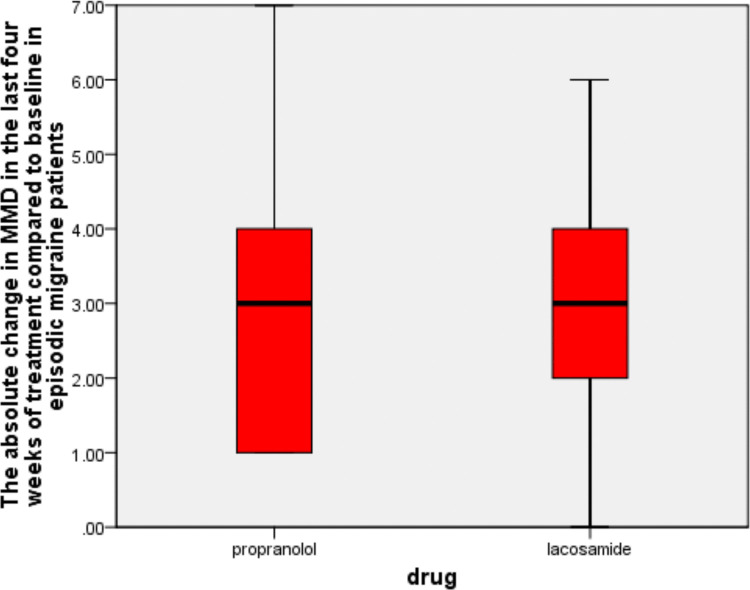
The absolute change in MMD in the last four 4 weeks of treatment compared to baseline in episodic migraine patients

We did not find any statistically significant difference between the two arms in the absolute change in MMD at 90 days (*P*-value = 0.13), as shown in Table 2 and Fig. 2.

Our trial showed that, at the 90-day mark. There was no statistically significant difference between the two arms in the percentage of participants who achieved ≥ 50% reduction in baseline migraine day frequency over the last 4 weeks of the treatment period (*P*-value = 0.23), as shown in Table 2.

**Table 2:** Analysis of efficacy outcomes in all patients

Clinical outcomes	Lacosamide arm (*n* = 300)	Propranolol arm (*n* = 300)	*P*-value
Primary efficacy outcome			
Absolute change in MMD in the last 4 weeks of the treatment period compared to baseline, (Median, IQR)†	3.0 (2.0–4.0)	3.0 (1.00- 4.0)	0.127
Secondary efficacy outcome			
Patients with ≥ 50% reduction in the baseline migraine days frequency in the last 4 weeks of the treatment period, no. (percentage)*	58.0 (19.3%)	70.0 (23.3%)	0.23
Absolute change in HIT-6 score in the last 4 weeks of the treatment period compared to baseline, (median, IQR)†	2.0 (2.0–4.0)	2.0 (1.0–5.0)	0.614
Absolute change in migraine days that required acute medications in the last 4 weeks of the treatment period compared to baseline, (median, IQR)†	3.0 (1.0–3.0)	3.0 (1.0–3*.0*)	0.572

†: Median (IQR, interquartile range), *: Percentage, **: Statistically significant at P value < 0.05 for the primary outcome and at adjusted P-value < 0.017 for the secondary outcomes MMD: monthly migraine days, HIT-6: Headache Impact Test-6

**Fig. 3:**
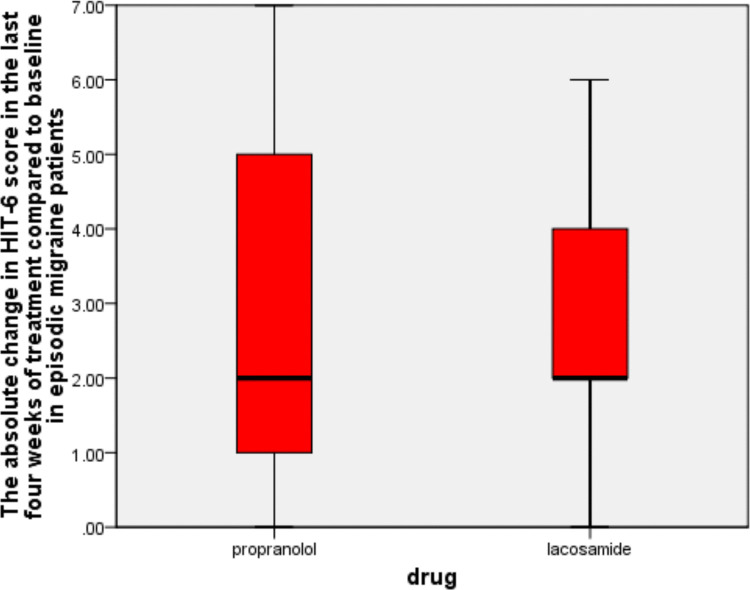
The absolute change in HIT-6 score in the last 4four weeks of treatment compared to baseline in episodic migraine patients

In addition, our trial showed that at the 90-day mark, the lacosamide arm showed a 2.66 ± 1.71-point reduction in HIT-6 scores compared to baseline (*P*-value = 0.001), while the propranolol arm showed a 2.81 ± 1.78-point reduction in HIT-6 scores compared to baseline (*P*-value = 0.001). We did not find any statistically significant difference between the two arms in the absolute reduction in the HIT-6 score at 90 days (*P*-value = 0.61), as shown in Table 2 and Fig. 3.

Moreover, we detected that, at the 90-day mark, the lacosamide arm showed a 2.26 ± 1.34-day reduction in the monthly migraine days that required acute medications compared to baseline, with a *P*-value of 0.002, while the propranolol arm showed a 2.45 ± 1.48-day reduction in monthly migraine days that required acute medications compared to baseline, with a *P*-value of 0.001. We did not find any statistically significant difference between the two arms in the decrease in monthly migraine days requiring acute medications at 90 days (*P*-value = 0.57), as shown in Table 2.

Regarding the analysis of the safety of treatment, we found that in the lacosamide group, 41 (13.7%) patients had side effects of which seven patients had palpitation, eight patients had back pain, two patients had hypotension, eleven patients had gastritis and vomiting, and four patients had diarrhoea, two patients had sleep disturbance, one patient had lethargy, two patients had chest tightness, four patients had numbness, and nine patients stopped treatment prematurely due to adverse effects, while in the propranolol group, 43 (14.3%) patients had side effects of which two patients had palpitation, three patients had back pain, eight patients had gastritis and vomiting, three patients had diarrhoea, five patients had sleep disturbance, four patients had lethargy, six patients had chest tightness, three patients had numbness, and eleven patients stopped treatment prematurely due to adverse effects, there were no statistically significant differences between the two groups apart from hypotension which was statistically significantly higher in the propranolol group (*P*-value 0.03), as shown in Table 3.

**Table 3:** Analysis of safety outcomes in all patients

Adverse effects, no. (percentage)*	Lacosamide arm (*n* = 300)	Propranolol arm (*n* = 300)	*P*-value
Gastritis	4.0 (1.3%)	3.0 (1.0%)	0.70
Vomiting	7.0 (2.3%)	5.0 (1.7%)	0.56
Diarrhoea	4.0 (1.3%)	3.0 (1.0%)	0.70
Palpitation	7.0 (2.3%)	2.0 (0.7%)	0.09
Bach pain	8.0 (2.7%)	3.0 (1.0%)	0.13
Hypotension	2.0 (0.7%)	9.0 (3.0%)	0.03**
Sleep disturbance	2.0 (0.7%)	5.0 (1.7%)	0.25
Lethargy	1.0 (0.3%)	4.0 (1.3%)	0.18
Chest tightness	2.0 (0.7%)	6.0 (2.0%)	0.16
Numbness	4.0 (1.3%)	3.0 (1.0%)	0.70

*Percentage, **Statistically significant at P-value < 0.05

## Discussion

Although migraine is a common neurological disorder with higher prevalence rates in the productive age and global burden of disease (GBD) 2010 has confirmed migraine to be one of the top causes of global health loss, its management is unsatisfactory due to the side effects of traditional treatment and the high costs of novel anti-migraine therapies [16, 17].

Anti-CGRP medications function by blocking the CGRP or its receptor and represent the newest options for migraine treatment and may be divided into gepants and monoclonal antibodies [6].

Anti-CGRP medications are still not widely available in developing countries owing to economic and logistical difficulties [6]. Therefore, we need to evaluate the potential of new oral medications for migraine preventive therapy.

Although propranolol is a class I migraine preventive beta-blocker [4], it has some contraindications in patients with atrioventricular block, heart failure, and bronchial asthma [7]. Moreover, antiseizure medicines such as topiramate and valproate, which are approved for migraine prevention, have many side effects and contraindications [18].

Although lacosamide has not been approved for the treatment of migraine, there have been some reports of its analgesic effects in animals and humans. In vivo models have demonstrated that lacosamide dose-dependently attenuated mechanical hyperalgesia following carrageenan injection and in rats suffering from Freund’s complete adjuvant-induced arthritis [19]. It also has been shown to suppress CGRP release from brainstem explants both under baseline conditions and following pharmacological stimulation, as well as to reduce the CGRP levels in the brainstem and trigeminal ganglia following nitroglycerin administration [10].

We aimed to evaluate the efficacy and safety of lacosamide as an alternative medication to propranolol for the prevention of migraine, especially in patients who cannot tolerate propranolol and approved antiseizure medications for migraine prevention.

We used propranolol in a dose of 80 mg Bid as the guidelines recommended using propranolol in migraine secondary prevention in a daily dose between 40 and 240 mg, and we followed the TOP-PRO study**,** which used propranolol in a daily dose of 160 mg [14]. In addition, we gradually increased the propranolol dose to minimize the incidence of side effects [20]. The dose of lacosamide 50 Bid was used following the only two trials that evaluated lacosamide in migraine patients [11, 21].

We found that in episodic migraine patients, the regular use of lacosamide yielded a reduction in the change in MMD in the last 4 weeks of treatment compared to baseline, a reduction in HIT score in the last 4 weeks of treatment compared to baseline, and a reduction in MMD that required acute treatment, which was comparable to these reductions produced by using propranolol.

Lacosamide could reduce the MMD as it resembled topiramate (a class I migraine preventive treatment [4] in blocking voltage-gated sodium channels (VGSCs) and inhibiting cortical hyper-responsiveness to stimuli; it has been shown to suppress CGRP release from brainstem explants both under baseline conditions and following pharmacological stimulation, as well as to reduce the CGRP levels in the brainstem and trigeminal ganglia following nitroglycerin administration in a rat model [10]. While a human study showed that the daily use of lacosamide 50 mg Bid for 3 months in episodic migraine patients was associated with a significant reduction in serum CGRP-LI [11].

In addition, the ability of lacosamide to reduce the HIT score agreed with many trials [22–25], which showed that using lacosamide in patients with neuropathic pain or trigeminal neuralgia was associated with improvements in the quality of sleep, patient global impression of change, quality of life, and pain interference owing to its sodium channel blocking properties and sodium channels are known to play a major role in pain signaling pathways [26].

Regarding the analysis of the safety of both treatments used in our trial, we found that there was no statistically significant difference in adverse effects between the two groups apart from hypotension, which was statistically significantly higher in the propranolol group, even though there has been no such study that compared the safety of lacosamide versus propranolol in migraine patients, our findings were in line with results of Yang et al. (2021), Hou et al. (2021), Shin et al. (2021), and Luk et al. (2012) who stated that lacosamide was well tolerated in epilepsy patients [8, 27–29], but in contrast to the findings of Domingues et al. (2009) and Chowdhury et al. (2021) who found that hypotension in migraineurs who received propranolol was not more frequent than in migraineurs who received nortriptyline or topiramate, respectively [14, 30]; we could explain this contradiction by our participants’ differences in genetics and ethnicity.

## Study Limitations and Conclusions

### Study Limitations

Our trial had some advantages, as it was the first blinded RCT worldwide to compare lacosamide versus propranolol in migraineurs. However, it had some limitations: the relatively small sample; second, all the patients were Egyptian, so we need to pursue a larger-scale randomized trial powered for both safety and efficacy to establish the validity and generalizability of the results.

## Conclusion

In episodic migraine patients, the regular use of lacosamide 50 mg Bid for 3 months yielded reductions in the monthly migraine days, migraine days that required acute medications, and HIT6 score comparable to those achieved using propranolol 80 mg Bid. Lacosamide was well-tolerated by migraine patients.

## Supplementary Information

Below is the link to the electronic supplementary material.

ESM 1 (DOCX.70.7 KB)

## Data Availability

The datasets generated and analyzed during the current study are not publicly available due to the ethical regulations of our university. However, they are available from the corresponding author (Mohamed G. Zeinhom) on reasonable request.
