# Supplementary material for: Lacosamide Versus Propranolol in Episodic Migraine, a Randomized Controlled Double-blinded Trial
Source: Mol Neurobiol. 2026 Mar 21;63(1):512. doi: 10.1007/s12035-026-05756-5 (PMC13005819; doi:10.1007/s12035-026-05756-5)
Supplement: Supplementary file 1 — (DOCX.70.7 KB) [file 12035_2026_5756_MOESM1_ESM.docx]

**Supplementary material**

**Eligibility criteria:** The study recruited participants between the ages of 18 and 65 who were diagnosed with episodic migraines following ICHD-3 (11),

All the patients received no migraine preventive treatment during the last month. The following conditions were considered ineligible for inclusion in our study: major neurological conditions such as (primary headaches other than migraine, stroke, epilepsy, and brain tumors, as well as patients with major systemic diseases such as malignancy, liver cell failure, renal failure, patients who received any migraine preventive treatment in the last month pregnant, and lactating patients, patients who had cardiovascular diseases as heart failure, ischemic heart disease, and cardiac arrhythmia, hypersensitivity to lacosamide or propranolol, or any other contraindications to the study medication. (9)

**Interventions:** We randomly assign 600 patients to receive lacosamide or propranolol, all of our patients underwent routine laboratory tests and MRI brain imaging.

Six hundred patients underwent randomization and underwent routine laboratory tests (fasting, postprandial blood sugar, and HBA1C, renal functions, liver functions, coagulation profile, complete blood count), ECG monitoring, transthoracic echocardiography, MRI brain (T1W, T2W, FLAIR).

We diagnosed hypertension when the systolic blood pressure (BP)≥140 mm Hg or diastolic (BP)≥90 mm Hg was detected on 2 recordings taken 5 min apart within 24 hours. (12,13)

The patient was classified as having diabetes when his fasting blood glucose level was higher than 126 mg/dL, and/ or casual plasma glucose was more than 200 mg/dL, and/or HbA1C was more than 6.5. (14,15)

The study had two parallel groups: the (A) group, which consisted of 300 patients who received (lacosamide 50 mg once daily for one week, then twice daily from the 8th day till 90th day) (9); the (B) group which consisted of 300 patients who received (propranolol 40 mg twice daily for one week then 80 mg twice daily from 8th day till 90th day) (16).

We used the Headache Impact Test-6 (HIT-6) to assess the burden of headache in each group; the HIT-6 consists of six items: pain, social functioning, role functioning, vitality, cognitive functioning, and psychological distress; the patient answers each of the six related questions using one of the following five responses: "never," "rarely," "sometimes," "very often," or "always." These responses are summed to produce a total HIT-6 score that ranges from 36 to 78, where a higher score indicates a greater impact of headache on the daily life of the respondent. It has four impact grades: little-to-no impact (HIT-6 score: 36-49), moderate impact (HIT-6 score: 50-55), substantial impact (HIT-6 score: 56-59), and severe impact (HIT-6 score: 60-78). (17,18)

Our investigation was explicitly intended to serve as a pilot study to evaluate the potential role of lacosamide in migraine prevention by comparing its efficacy and safety with a well-established migraine preventive treatment such as propranolol, as well as to determine whether it would be feasible to proceed with a large-scale randomized clinical trial that would be sufficiently powered to evaluate the safety and effectiveness of lacosamide in migraine prevention.

Our trial followed most of the IHS guidelines for trials for migraine prevention; our trial was a randomized, blinded study, we defined migraine according to ICHD criteria, we excluded participants with other headaches, our patients had at least four migraine attacks per four weeks, our patients had migraine for at least 1 year prior to entering into the study, we followed up our patients for three months, our patients were 18 years or older, we included both males and females, our patients did not meet the criteria of medication overuse headache, we excluded patients who had hypersensitivity towards the trial medications, and we followed up our patients every four weeks. (19)

**Randomization and Blinding**

Our study was a double-blinded trial; an independent statistician generated a blocked randomization sequence using computer-generated random numbers with a block size of four, and all the investigators included in the study did not know the block size, in a one-to-one ratio, participants were randomly assigned to receive either propranolol or lacosamide by a specially trained and qualified nurse. All the investigators included in the study did not know the patients' assignments. We prepared sequentially numbered opaque Pill boxes and 600 labels for each drug, drug A or B. According to the randomization chart, we attached the number to the box. Patients were recruited sequentially and were given enrollment numbers starting from 1, which were mentioned in their files. Files carrying the same number as the patient enrolment number were then opened, and the patients were assigned to receive drugs A or B based on their randomization. Drug A included lacosamide 50 mg tablets, and Drug B included propranolol 40 mg tablets.
